# Current cardiac imaging techniques for detection of left ventricular mass

**DOI:** 10.1186/1476-7120-8-19

**Published:** 2010-06-01

**Authors:** Aksuyek S Celebi, Hulya Yalcin, Fatih Yalcin

**Affiliations:** 1 Cardiology Department, Tokat State Hospital, Tokat, Turkey; 2 Johns Hopkins University School of Medicine, Cardiovascular Imaging, Division of Cardiology, Department of Medicine, Baltimore, USA

## Abstract

Estimation of left ventricular (LV) mass has both prognostic and therapeutic value independent of traditional risk factors. Unfortunately, LV mass evaluation has been underestimated in clinical practice. Assessment of LV mass can be performed by a number of imaging modalities. Despite inherent limitations, conventional echocardiography has fundamentally been established as most widely used diagnostic tool. 3-dimensional echocardiography (3DE) is now feasible, fast and accurate for LV mass evaluation. 3DE is also superior to conventional echocardiography in terms of LV mass assessment, especially in patients with abnormal LV geometry. Cardiovascular magnetic resonance (CMR) and cardiovascular computed tomography (CCT) are currently performed for LV mass assessment and also do not depend on cardiac geometry and display 3-dimensional data, as well. Therefore, CMR is being increasingly employed and is at the present standard of reference in the clinical setting. Although each method demonstrates advantages over another, there are also disadvantages to receive attention. Diagnostic accuracy of methods will also be increased with the introduction of more advanced systems. It is also likely that in the coming years new and more accurate diagnostic tests will become available. In particular, CMR and CCT have been intersecting hot topic between cardiology and radiology clinics. Thus, good communication and collaboration between two specialties is required for selection of an appropriate test.

## Introduction

Left ventricular hypertrophy (LVH) is an unfavorable condition, which is consistently and strongly associated with significant cardiovascular morbidity and mortality [1]. In addition, left ventricular (LV) mass portends poor patient prognosis independent of traditional risk factors [2]. Furthermore, numerous therapeutic agents are observed to promote LV mass reduction in parallel with improved clinical outcome [3]. As a result, LV mass is suggested as a regular study finding of any cardiac imaging modality [4].

LV mass determination by various imaging modalities is principally based on shell volume, which is obtained as the difference of epicardial and endocardial volumes. Shell volume is subsequently converted to mass by multiplying with the specific density of myocardial tissue (usually taken as 1.05 g/mL).

Individual variation in body size influences the assessment of LV mass. Indexing is of importance to differentiate between health and disease, to assess the severity of disease, to allow comparisons among varying body size, and to recognize alterations over time. LV mass may be expressed as an index for some variables of body size. The best method for LV mass indexing is still under debate. Different methods of indexation result in considerably different prevalence of LVH [5]. Some investigators have traditionally indexed LV mass to body surface area [6]. However, indexing by body surface area may inappropriately normalize LV mass and must be critically questioned [7]. Normalization for the allometric power of its relation to body height (height^2.7 ^) provides a greater attributable risk than other methods of indexation [8]. Indexing to height in meters to the power 2.7 successfully facilitates identification of LVH among obese subjects, as well [9]. Nevertheless, lean body mass was found to be more ideal than height and body surface area for indexing LV mass (7). Along with these, identification of LV mass needs to take into account the influence of different ethnic groups and sexes [6].

The accurate measurement of LV mass is difficult, partly due to the oblique angle between heart and chest, continuous movement of the heart, and the lack of a proper technique for imaging the whole left ventricle. Initial measurements with electrocardiography (ECG) data were insensitively and nonspecifically surrogate markers for LVH. Imaging modalities have overwhelmingly superseded the ECG over time. Plenty of diagnostic imaging modalities have been used to asses LV mass. Unfortunately, LV mass evaluation has been underestimated in clinical practice, thus not taking full benefit of the acquired data. We are going to discuss echocardiography, cardiovascular computed tomography (CCT), cardiovascular magnetic resonance (CMR), single photon emission computed tomography (SPECT) and contrast ventriculography (CV) for LV mass assessment. It is to be known that none of the imaging modalities is actually performed for only LV mass assessment.

## Echocardiography

The past few decades have seen echocardiography evolve into a cornerstone of modern cardiac investigation. Gradually, various ultrasound-based techniques with burgeoning technologic improvements have been invented, investigated, and then integrated. Image quality has become better, often generating images comparable with with those of CMR. Hence, echocardiography has been used essentially to provide mechanistic insights on cardiac morphology such as LV mass. Routine calculation of LV mass should be an integral part of an echocardiographic examination [10]. Echocardiography is not only of use in assessing LV mass but also of use in demonstrating reduction in LV mass after therapy [3]. Echocardiography constitutes to be the first choice cardiac imaging modality for assessment of LV mass. Moreover, echocardiography may further display LVH distribution pattern.

Motion-mode (M-mode), 2-dimensional echocardiography (2DE) and finally 3-dimensional echocardiography (3DE) attract attention for LV mass assessment.

## M-mode Echocardiography

**Figure 1:**
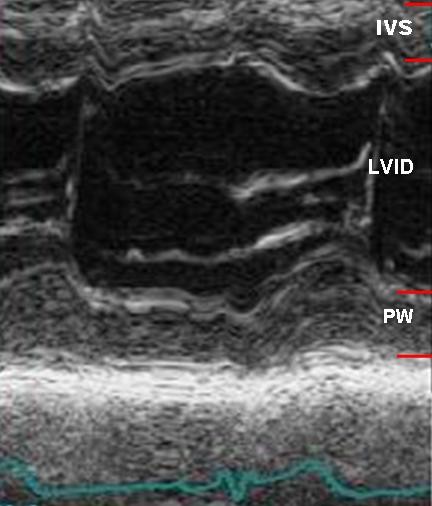
**M-mode left ventricular measurements are obtained in parasternal long axis from leading edge to leading edge technique**.

**Figure 2:**
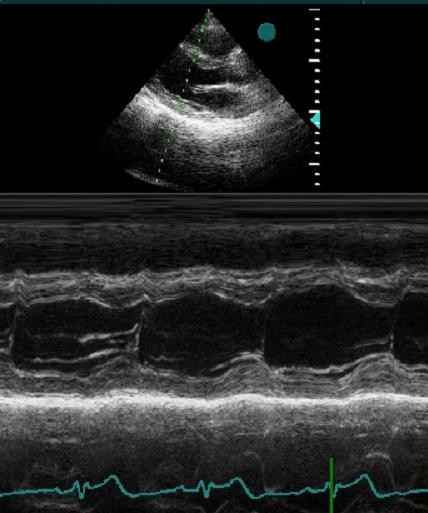
**M-mode cursor position is perpendicular to interventricular septum and posterior wall of the ventricle at the mitral valve chordae level, reflecting minor-axis dimension**.

First echocardiographic modality that is to be offered for LV mass evaluation is M-mode echocardiography. M-mode echocardiography was one of the earliest modalities of echocardiography to come into clinical use. This modality records the position and motion of echoes arising from intracardiac structures with regard to time, producing one-dimensional information. LV mass detection and quantification can be clearly estimated from linear measurements by employing American Society of Echocardiography (ASE) recommendations (figures 1 and 2) [11]. LV mass by M-mode was in close correlation with necropsy, reinforcing this method [12].

LV mass is calculated by cubing formula, which assumes that LV is represented by prolate ellipse. M-mode echocardiography is useful for quantitating LV mass when ventricles are geometrically uniform for this reason.

Thanks to its wide availability, moderate expense, anatomic and prognostic validation, and lack of radiation, contrast agent, or claustrophobia, M-mode echocardiography has been used widely for estimating LV mass in clinical trials [13,14]. Another useful aspect of M-mode imaging is its easily analyzable depiction of continuous motion versus time with high temporal resolution, which makes it useful for timing of cardiac events. M-mode also yields good interface definition of LV chamber and this has been further improved by 2D guidance.

**Figure 3:**
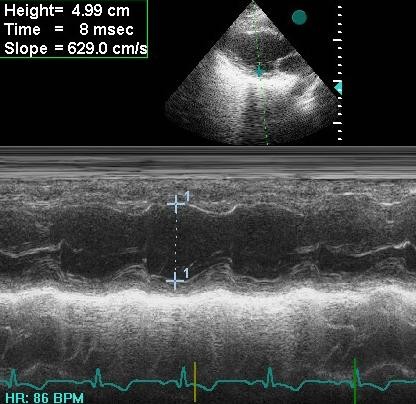
**Minor-axis dimension is overestimated due to tangential measurement in M-mode echocardiography (4.99 cm)**.

**Figure 4:**
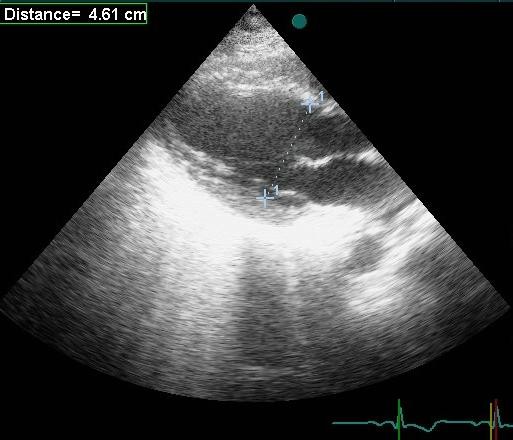
**The true minor axis is obtained from 2-DE (4.61 cm)**.

Although measurement of LV mass using M-mode has proved to be the most efficient method and widely used in clinical and research, there are definite disadvantages of this method such as need for geometric assumption. This assumption may lead to inaccuracy that may occur with altered shape of heart, for example, aneurysm, hypertrophy, and wall motion abnormalities [15]. Since LV mass is estimated by the geometric cubing formula, small errors in these measurements are amplified to the third power. Secondly, it may not always reflect the true minor-axis dimension of LV in parasternal long axis (figures 3 and 4). An optimally oriented M-mode cursor by 2D guidance may be beneficial in this setting [10]. If 2D guidance is not available, direct 2D measurements may be alternatively substituted and is considered more accurate [10,16].

In other words, M-mode method is reasonable to use for normally shaped ventricles, but it is likely to give erroneous results in abnormally shaped ventricles. However, this method is presently the only one recommended in a guideline for determining LVH as subclinical organ damage [17].

## 2-Dimensional Echocardiography

The second modality of echocardiography for LV mass evaluation is 2DE. LV mass can directly be computed by 2D linear measurements and standards have been established by ASE to support synergy in measurments [10,16,18]. LV mass can also be calculated from planimetered dimensions of 2D images obtained during real-time transthoracic imaging with the area-length or truncated-ellipsoid formulas. This specific methodology for 2D estimation of LV mass has good agreement with autopsy [19].

Utilization of 2DE overcomes the common problem of oblique parasternal images that result in overestimation of cavity and wall dimensions from M-mode echocardiography. 2DE is also preferred to M-mode echocardiography in the case of regional wall motion abnormalities, because M-mode modality identifies the function of the basal segments only rather than whole LV segments. Thus, 2DE has widely supplanted isolated M-mode linear measurements.

Some limitations are also encountered with this modality. A difficult echo window usually does not let to obtain accurate delineation of endocardial borders. This confinement hinders quantification of LV mass. This has largely been resolved by administration of a contrast agent, which is reported to be safe [20]. However, the risk of serious cardiopulmonary reactions of contrast agent is highlighted and it is recommended to closely monitor high-risk patients with pulmonary hypertension or unstable cardiopulmonary conditions [21]. Patients with severe intracardiac shunt or hypersensitivity to the agent are absolute contraindications to emphasize [20]. While using 2D linear measurments, ultrasound interrogation angle should be perpendicular to the endocardium as much as possible and length of LV should be maximized for more accurate LV mass measurement [3]. Finally, it is operator-dependant, thereby requiring some standards to improve reproducibility and quality of studies [5,22]. A core laboratory with sufficient experience may also help determining better reproducibility and decreasing operator variability, especially when employed in clinical trials [6]. In contrast to other techniques, it interactively requires a strong cerebral-to-arm connection and vice versa throughout the examination.

In conclusion, an optimal method for LV mass by echocardiography should be safe, rapid, reliable, cost-effective, and widely applicable in order to be utilized routinely in a busy clinical laboratory. 2DE remarkably seems to fulfill these criteria.

## 3-Dimensional Echocardiography

3DE should be the next logical step for assessment of LV mass. Newer matrix array transducers allow real time 3-dimensional (3D) visualization of entire heart by acquiring a pyramidal image. By means of this, 3DE is a novel use of echocardiography to display the 2 dimensional imaging planes of cardiac structures within the 3D data set.

Recently, 3DE has demonstrated incremental value about LV mass over conventional echocardiographic modalities (M-mode, 2DE) in different patient populations [18,23]. The major strength of 3DE is the geometric assumption free accurate imaging of cardiac structures. As mentioned before, conventional echocardiographic modalities are limited mainly by inaccuracies and variations caused by use of geometric assumptions in case of asymmetric LV geometry and unintended use of oblique planes. 3DE, based on the assessment of a larger number of tomographic views, resolves these limitations. 3DE correspondingly has the advantage of reducing dependence on geometric models and reducing error based on angulated images [6,24]. Thus, 3DE offers LV mass determination substantially comparable to magnetic resonance imaging, as for global LV volumes [25]. This correlation between 3DE and CMR has also been stronger than that has been between conventional echocardiography and CMR [26]. Interobserver variability of real-time 3 dimensional echocardiography (RT3DE) was additionally reported to be significantly lower than that of 2DE, supporting its superiority [27]. This superiority has already been anatomically in agreement with autopsy findings [24]. 3DE and CMR also produce comparable results in terms of LV mass with similar interobserver variability [28]. As 3DE is less costly and more attainable, it is modality of choice for serial follow-up.

Besides, 3DE may provide comprehensive evaluation of the cardiovascular structure in addition to LV mass evaluation, which overcomes the limitations of conventional methods [29]. 3DE also shows promise in the assessment of congenital heart disease, thereby influencing clinical management and perhaps obviating the need for CMR in some cases [30]. Moreover, 3DE is less dependent on operator practice and help to compensate operator inexperience when compared to conventional echocardiography.

3DE was previously reported to cumbersome, time-consuming, and difficult to perform compared to conventional methods. These technical complexities have limited its widespread application in clinical practice and research [23]. Despite these drawbacks, clinical use of 3DE is recently urged by the marked reduction in acquisition time and the unique possibility of on-line rendering on the ultrasound system. Moreover, the time expenditure for 3DE using the semiautomatic algorithms was similar compared with that for 2DE [15]. The time needed for the acquisition and analysis of RT3DE data were further acceptable in another report [31]. It has even been offered as an accurate and time-saving option in clinical practice [24].

Care must be taken to include the entire LV cavity within the pyramidal scan volume. 3DE provides accurate LV mass when entire LV is covered in the 3D pyramid [24]. Consequently, 3DE is not yet clearly suited for the assessment of patients with severe dilated cardiomyopathy, ventricular aneurysms, or ventricles with heavy trabeculae as it may not be possible to display point of interest structure into the pyramidal scan volume. Therefore, there needs to be further studies to define better the applicability of this technique in these populations.

3DE echocardiography allows perspectives not achievable conventionally. 3DE is feasible in the clinical setting and provides fast and accurate assessment of LV mass, which is superior to conventional echocardiographic methods, especially in dsitroted hearts. This leads to greater sensitivity and reduced sample size. The recent advances in the transducer and computer software technology may further improve the accuracy and reproducibility of the technique. With increasing clinical experiences in 3DE, visualization and quantitation of cardiovascular structure and function will improve.

## Cardiovascular Computed Tomography

Computed tomography (CT) is an emerging modality for the noninvasive assessment of cardiac anatomy and function. It is named as CCT by a recent statement for cardiovascular field and this represents a specialized use of CT [32]. Multidetector CT (MDCT) has improved temporal and spatial resolutions, thereby allowing successful evaluation of coronary anatomy and cardiac morphology. State of the art CT practice is capable of concomitant LV mass assessment. Besides, there has been a high correlation for LV mass evaluated by CCT and CMR, so that CCT is offered as a strong alternative for patients with contraindications to CMR [33]. This finding was confirmed in another study comparing CT with echocardiography [34]. In the light of these data, LV mass estimation has been encouraged in a routine coronary CT angiography report at present [35].

Semiautomatic analysis software has simplified endocardial and epicardial contour detection in a significantly shorter period with the same reliability. Despite this, this algorithm overestimated LV mass compared with manual contour tracing [36]. On the other hand, CCT with semiautomatic software is still reported to accurately quantify LV size and function including LV mass compared to CMR [33]. Objective quantification of LV function using semiautomatic software analysis is feasible, accurate, and time-effective.

Patients with heart failure and distorted ventricles seem to benefit the most from CCT to elucidate etiolgy. Because information in terms of LV function or mass by CCT, complement already acquired data concerning coronary anatomy [37]. Thus, CCT is preferred to CMR in view of its ability to provide superb demonstration of coronary anatomy and to give excellent information about LV mass at the same time in these patient groups [38].

To best of our knowledge, there is no head-to-head study comparing all modalities just for LV mass measurement. Nevertheless, CCT seems to be a reasonable method to evaluate LV function in well agreement with CMR and because of superiority over echocardiography, CV, and SPECT [39,40]. Despite good correlation with respect to LV mass, 64-slice CCT was preferable to CMR owing to better sensitivity and specificity for akinetic segments in patients with acute coronary syndrome. In contrast, 16-slice CCT was unable to repeat this result [41].

Although CCT entails many benefits, some limitations are not negligible. Primary disadvantage is the inevitable radiation exposure called as stochastic effect. In general, radiation exposure is estimated to be between 7 and 21 mSv depending on the generation of the CCT and the use of radiation-reducing algorithms [42]. This raised the concerns about lung cancer and breast cancer risk for repetitive use in daily practice, particularly in younger people or women of childbearing age [43]. Unfortunately, technical factors that enhance image quality result in a higher radiation dose. Efforts to reduce the radiation dose in CCT, which are not our present issue in this review, has been discussed in depth [32,39,44]. Moreover, a science advisory has been published regarding with ionizing radiation in cardiac imaging and diligently conveys newest recommendations for cardiologists [45].

Contrast medium induced nephropathy is a caveat for CCT in clinical practice. Calculating a simple risk score for patient selection and new preventive pharmacological measures are comprehensively discussed in a recent report [46]. Another caveat for contrast agent is allergic reactions, including severe anaphylaxis. Recommendations for handling these problems are debated previously [47]. Thus, the need for iodinated contrast makes CCT relatively contraindicated in patients at risk for contrast-induced nephropathy or those with a history of allergy to contrast media. Of interest, successful efforts concerning LV mass detection with noncontrast CT as well as CMR should also be realized to solve this problem [48].

High heart rate renders scan incompletely interpretable with poor image quality because of motion artifacts. Beta-blockers are usually administered prior to CT for heart rate control and reducing motion artifacts, thus improving image quality. Directions for patient preparation and beta-blocker administration are previously discussed in depth previously [47]. There was concern that beta-blockers would have some impact on left ventricular functional parameters. Nevertheless, administration of beta-blockers is found to not affect LV mass assessment [49]. Despite well tolerance to beta-blockers, this may cause cautious observation and a prolonged stay of the patient, which is unwelcomed. Instead, a segmental reconstruction algorithm with CCT was alternatively documented to be accurate and correlated well with CMR over a broad range of heart rates without using beta-blockers [39].

Cardiac rhythm is another determinant for better image quality. Since MDCT angiography requires electrocardiogram-gated acquisition and reconstruction from several cardiac cycles, it is presently limited to patients with stable, regular heart rates. Irregular cardiac rhythm makes images suboptimal and even non-diagnostic.

Obesity represents another challenge for CCT imaging. Image resolution may be deteriorated in morbidly obese patients because of x-ray attenuation. It is not yet clear how CT should be integrated in the clinical practice for patients with higher body mass index, who unfortunately represent an increasingly prevalent segment today.

Given the predominant disadvantages of contrast media application and radiation exposure, performing CCT only for analysis of LV mass seems not reasonable. Moreover, new generation CT such as 64-slice CT and dual source-CT seems to enhance the clinical utilization because of increased temporal resolution and reduced acquisition time. Spatial resolution by contemporary CT machines already outperforms the spatial resolution by magnetic resonance (MR) imaging. This fortunately comes at no additional cost of radiation dose or contrast media delivery, as LV mass data is already delivered from acquired data of CT coronary angiography.

## Cardiovascular Magnetic Resonance

MR imaging is one of the newest and most exciting imaging techniques in the cardiovascular armamentarium. Official name is accepted as "cardiovascular magnetic resonance" by the Society for Cardiovascular Magnetic Resonance and declared in a statement when it is applied to heart alone or heart and peripheral vascular structures [32]. CMR has recently moved from a diagnostic tool mainly used for congenital heart disease, large vessels, pericardium, and tumors to a clinically proven, safe, and comprehensive imaging modality with a broad range of indications including LV mass quantification. Its appropriateness has been approved in order to assess LV mass [50].

LV is not subject to any geometric assumption by CMR. Major advantage of CMR is accuracy and reproducibility by using 3D approach. Therefore, CMR is considered the reference standard for both baseline and serial LV mass measurements [11,13,51,52]. Furthermore, this technique enables a reduction in sample size in clinical trials [51,52]. LV mass measurement by MR has eventually been in excellent agreement with autopsy [53].

Its versatility is unmatched by any other individual imaging modality. In addition to allowing for accurate anatomic information, it also provides functional information assisting in identifying patients at risk. Three-dimensional left ventricular geometric analysis using CMR yielded more accurate information about left ventricular geometry compared to conventional echocardiographic methods with higher reproducibility and lower variability [54,55]. CMR is also able to detect LVH in patients with seemingly normal echocardiographic results [56]. This greater sensitivity is explained by geometric assumption-free quantification of LV mass by CMR.

LV mass measurement by CMR is very well suited to clinical examinations: it is noninvasive, does not expose the patient to ionizing radiation, and provides images of high temporal resolution and excellent soft tissue contrast without the need for contrast medium injection. Some other advantages are superior quality of images mostly not limited by poor acoustic windows or operator inexperience compared to echocardiography. Although 3DE removes the limitation of geometric assumption, it disappointingly falls short of the limitation of poor acoustic window. When echocardiography is impractical in patients with poor acoustic windows, CMR permits cardiac structures to be imaged.

One of the end organ damages resulting from longstanding hypertension (HT) is LVH. Detecting LVH is strongly recommended in HT guidelines, as well [57]. Another objective is to detect remediable causes of HT in guidelines. CMR is capable of demonstrating secondary causes owing to availability of tissue characterization and angiography along with LV mass assessment [58]. Hence, CMR may be beneficial as a further comprehensive investigation modality when there is a suspect of secondary HT, especially for aortic coarctation, renal artery stenosis and surrenal gland related pathologies.

CCT suffers the burden of ionizing radiation and contrast medium that makes it less than ideal. Unlike CCT, CMR is free of ionizing radiation or injection of potentially nephrotoxic contrast medium. CMR can therefore be repeated as often as necessary for follow-up and an eligible alternative method to CCT (28). Despite previously mentioned superiority of CCT about differentiation of heart failure etiology, CMR may be an alternative and guide therapy as long as CCT is contraindicated or avoided [59].

In addition to presence and magnitude of LVH, its distribution can be determined by 2DE. Notwithstanding this positive effect, CMR has been competitive with 2DE respecting this issue. CMR is known to be highly more effective in describing the degree, diversity, and extension of LVH [60]. Apical views may also be exclusively suboptimal in quality by 2DE. CMR is reported to demonstrate apical region precisely and to be more accurate in the diagnosis of apical hypertrophy than 2DE [61].

Myocardial fibrosis (MF) has reasonably known to be associated with an increased risk of ventricular arrhythmias [62]. Unique to CMR over other imaging modalities is the characterization of tissue in high spatial resolution; thereby detection of MF has become available by CMR. This is an additional prognostic marker in addition to LV mass in risk stratification. Thus, it makes CMR one-step forward compared to other modalities.

Despite these promising results, several important limitations of CMR should be kept in mind. It continues to have problems of cost, limited availability, and lack of portability. These limitations avert to use CMR routinely.

Contraindications for CMR traditionally exist with ferromagnetic metal devices such as a pacemaker or cardiac defibrillator [52]. There are some justified reasons for that; 1) There is potential risk of arrhythmias due to induced lead currents, 2) The tips of the leads can get extremely hot causing tissue damage, which can potentially cause lead dysfunction, 3) It is also noticed that ferromagnetic metallic implants are liable to mechanical pull and rotation of the device, 4) Image artifacts from metallic implants may also preclude quantitative analysis. In addition to these, concerns about implantable devices have centered on the potential of CMR to inhibit the function of the device and change programming. The American Heart Association and the European Society of Cardiology justifiably have issued statements on this topic before [63,64]. According to these statements, CMR may be considered after careful evaluation in selected patients, only done when clinically indicated in the absence of an alternative imaging modality, and the diagnostic benefit from MR must outweigh the presumed risks. In addition to these measures, new MR compatible pacing systems are designed to confirm the safety and efficacy in the MR environment and to probably remove these challenges. The interim results of a recent study of these devices are fortunately encouraging [65].

Claustrophobia becomes a remarkable problem in some patients. Mild sedation may handle the limitation of claustrophobia [51]. Open magnets also appear to eliminate this limitation in the near future.

Another criticism for CMR has been duration of analysis to obtain LV mass data. Now, faster semiautomatic border definition procedures seem to make time problem unlikely as for CT and 3DE [51]. This procedure provides accurate and quick assessment of LV mass. Recent breakthroughs in CMR technique can supersede repeated breath holding for analysis and help shorten acquisition time [66,67].

One potential drawback of CMR may be the increased risk of nephrogenic systemic fibrosis (NSF) by virtue of gadolinium in acute and chronic renal failure [68]. In these cases, gadolinium-based contrast agents should be avoided unless it is essential and other imaging modalities should be preferred. FDA has requested that the manufacturers include a new Boxed Warning and new warnings section in the labels that describe the risks of developing NSF.

CMR provides a noninvasive, accurate, and reproducible LV mass assessment without radiation, contrast agent, and geometric assumption. The clinical role of magnetic resonance in diseases of the heart and great vessels is rapidly evolving. Besides, some applications of MR to the cardiovascular system are now established components of a cardiac workup. Despite these benefits, CMR has yet to fill this role because of cost, availability, and more minor issues regarding device incompatibilities and patient tolerance for routine clinical use. Furthermore, most studies have used 1.5 T magnets. Clinical 3-T MR systems are becoming widespread and their role awaits further investigation.

## Single Photon Emission Computed Tomography

SPECT is another imaging modality for LV mass assessment. SPECT, using a variety of protocols and with either thallium-201 or technetium-99 m tracers can be used for gated perfusion purposes. Thus, SPECT should particularly be favoured in patients with suspected or known coronary artery disease.

Several limitations of SPECT merit attention. Firstly, this technique is rather expensive and becomes impractical when serial evaluation is essential. Secondly, it inherently involves radiation and 85% of the radiation of nuclear medicine studies arises from cardiac imaging modalities [51,69]. Radiation exposure is estimated to be approximately 9 mSv for ^99 m^Tc stress/rest and 41 mSv for ^201^Tl stress/rest [42]. As discused before, recommendations for the safe use of cardiac imaging with radiation exposure are discussed in depth [45]. Cumulative radiation dose from SPCET for multiple follow up pose a problem for long-term follow up. Therefore, echocardiography or CMR, which can be performed repeatedly, may be a rational option in this setting. Thirdly, partial volume effect and limited image resolution may lead to inaccurate measurement in the event of small heart [51]. Fourth limitation is the presence of large perfusion defects disabling calculation of LV mass. Finally, it is subject to low spatial and temporal resolution [51].

SPECT permits perfusion assessment of myocardium and LV function encompassing LV mass within a single study. However, SPECT, with taking into account its inferiority to other imaging modalities for LV mass assessment, should not be standard.

## Contrast Ventriculography

CV allows estimation of LV mass along with coronary anatomy. Although CV was introduced to clinical practice before above-mentioned methods, it was left under used and neglected in due course. This forgotten method was once called as the reference method for LV mass evaluation [70]. Calculation of LV mass using CV was in accordance with actual weights in postmortem human hearts [71]. This method may decrease downstream testing costs, such as echocardiography, CCT, and CMR. Therefore, CV may possibly confer the most cost-effective modality for LV mass assessment in patients undergoing left heart catheterization [72].

This method is subject to some limitations. Pericardial diseases such as effusion or thickening may invalidate this method. RV hypertrophy may also lead to erroneous measurement of LV mass. Pulmonary lesions that obscure left heart border additionally may preclude assessment of LV mass with this method. Suboptimal opacification will make accurate LV mass measurement unlikely. If lateral wall does not appear to be representative of whole LV (aneurysm, asymmetric LVH), this method may give unreliable measurement. Its invasiveness, radiation exposure, and contrast media are finally other points of concern disabling it in clinical practice.

## Conclusion

Conventional echocardiography currently continues to be the imaging modality of choice for the assessment of LV mass in routine clinical practice. Unfortunately, conventional echocardiography has been marred by many limitations in spite of advancements in hardware and software technology. Evaluation of LV mass is now feasible by various diagnostic imaging modalities other than conventional echocardiography. CMR evenly offers optimal and reference standard for LV mass assesment. CCT and 3DE are comparatively still experimental, whereas SPECT and CV seem to fall in oblivion. However, before extensive use, these methods need to be thoroughly tested in larger studies and validated in distinct patient populations. Every patient should be considered in his or her own condition due to lack of established guidelines in terms of LV mass assessment. All modalities need to be taken into account in the context of their specific advantages and disadvantages (table 1). The near future holds major developments that may eliminate some of the disadvantages. Good communication between cardiologist and radiologist is becoming obviously crucial to track the appropriate use of imaging tests in clinical practice.

**Table 1:** Comparison between different techniques.

Technique	Advantages	Disadvantages
M-mod echocardiography	-safe, -rapid, -cost-effective, -available, -portable, -highly versatile, -noninvasive	-limited in patients with poor acoustic image (obese and chronic obstructive pulmonary disease) and asymmetric LV -operator dependent

2DE	-safe, -rapid, -cost-effective, -available, -portable, -highly versatile, -noninvasive	-limited in patients with poor acoustic image (obese and chronic obstructive pulmonary disease) and asymmetric LV , -operator dependent

3DE	-safe, -rapid, -cost-effective, -available, -portable, -versatile -highly versatile, -noninvasive	-limited in patients with poor acoustic image (obese and chronic obstructive pulmonary disease)

CMR	-safe -highly versatile, -noninvasive -reproducible	- Limited in claustrophobic patients, acute and chronic renal failure or those with metallic implants, -long testing time, -expensive, -not portable, -limited availability

CCT	-medium versatile, -noninvasive	- Limited in patients with arrhythmia or obesity, -long testing time, -radiation exposure, -limited for serial studies, -not portable, -contrast agent exposure

SPECT	-medium versatile, -noninvasive	-long testing time, -radiation exposure, -limited for serial studies, -not portable, -expensive

CV	-medium versatile,	-invasive, -radiation exposure, -not portable, -contrast agent exposure, Limited in patients with pulmonary, and pericardial disease

## Competing interests

The authors declare that they have no competing interests.

## Authors' contributions

All authors contributed to the paper and meet the criteria for authorship. All authors read and approved the final manuscript.
